# The Increased Expression of HLA-DR and ICAM-1 Molecules by Human Bronchial Epithelial Cells, Induced by Activated Mononuclear Cells, is Downregulated by Nedocromil Sodium

**DOI:** 10.1155/S0962935194000682

**Published:** 1994

**Authors:** O. Sacco, S. Lantero, L. Scarso, V. Frangova, V. Ottolini, G. A. Rossi

**Affiliations:** 1 Divisione di Pneumologia Istituto G. Gaslini Largo G. Gaslini 5 Genova 16148 Italy; 2 Centro di Immunoematologia e Servizio Trasfusionale Istituto G. Gaslini Largo G. Gaslini 5 Genova 16148 Italy

## Abstract

To test the hypothesis that mononuclear cell products could increase the expression of HLA-DR and ICAM-1 molecules in bronchial epithelial cells (BECs), subconfluent cultures of human BECs, obtained from surgically resected bronchi, were incubated with PHA-activated blood mononuclear cell conditioned media (BCM-CM) or recombinant IFN-γ. The presence of HLA-DR and ICAM-1 molecules on BECs was then evaluated by specific antibody staining and flow-cytometry analysis. The addition to BEC cultures of different concentrations of PHA-stimulated BMC-CM, or of IFN-γ induced a dosedependent increase of HIA-DR and ICAM-1 expression, while no effect was observed with unstimulated BMC-CM. The ability of nedocromil sodium and, as control, of dexamethasone, to prevent the upregulation of HLA-DR and ICAM-1 expression on BECs was then tested. Increasing concentrations (10^−7^ to 10^−4^ M) of nedocromil significandy inhibited HLA-DR and ICAM-1 expression by BECs in a dose-dependent fashion. A similarly dose-dependent inhibitory effect was also observed with dexamethasone, which, however, was less active than nedocromil on HL-ADR expression and more active on ICAM-1 expression. Finally, nedocromil and dexamethasone showed a significant synergistic effect on the expression of both cell surface molecules at the lowest concentrations tested.


Mediators of Inflammation 3, S7–S13 (1994)

MOIa20

### The increased expression of HLA-DR and ICAM-1 molecules by human bronchial epithelial cells, induced by activated mononuclear cells, is downregulated by nedocromil sodium

**O. Sacco,[1] S. Lantero,[1] L. Scarso,[2] V. Frangova,[1] V. Ottolini[1] and G. A. Rossi[1,CA]**

[1]Divisione di Pneumologia and [2]Centro di Immunoematologia e Servizio Trasfusionale, Istituto G. Gaslini, Largo G. Gaslini 5, 16148 Genova, Italy

[CA] Corresponding Author

**To test the hypothesis that mononuclear cell products could increase the expression of HLA-DR and ICAM-1 molecules in bronchial epithelial cells (BECs), subconfluent cultures of human BECs, obtained from surgically resected bronchi, were incubated with PHA-activated blood mononuclear cell conditioned media (BCM-CM) or recombinant IFN-γ. The presence of HLA-DR and ICAM-1 molecules on BECs was then evaluated by specific antibody staining and flow-cytometry analysis. The addition to BEC cultures of different concentrations of PHA-stimulated BMC-CM, or of IFN-γ, induced a dose-dependent increase of HLA-DR and ICAM-1 expression, while no effect was observed with unstimulated BMC-CM. The ability of nedocromil sodium and, as control, of dexamethasone, to prevent the upregulation of HLA-DR and ICAM-1 expression on BECs was then tested. Increasing concentrations ($10^{-7}$ to $10^{-4}$ M) of nedocromil significantly inhibited HLA-DR and ICAM-1 expression by BECs in a dose-dependent fashion. A similarly dose-dependent inhibitory effect was also observed with dexamethasone, which, however, was less active than nedocromil on HLA-DR expression and more active on ICAM-1 expression. Finally, nedocromil and dexamethasone showed a significant synergistic effect on the expression of both cell surface molecules at the lowest concentrations tested.**

**Key words**: Adhesion molecules, Bronchial epithelial cells, Cromones, HLA-DR, ICAM-1, Interferon-γ

### Introduction

The airway epithelium, as well as acting as a barrier between the outside and inside environment, also appears to have important metabolic and immunological properties.[1] In this context, bronchial epithelial cells can actively release a range of mediators which participate in inflammatory[2] and healing[3] processes and which modulate immune responses within the lung.[4] The development of any inflammatory response depends upon efficient antigen presentation to relevant leukocytes, the mobilization of these cells to the inflammatory site, and their attachment to the target cells, all of these steps being dependent on cell-to-cell contact. A basic mechanism for cellular interaction involves the expression of class II human leukocyte antigens (HLA), such as HLA-DR. These major histocompatibility (MCH) class II gene products have a functional role in guiding helper T-cells in the recognition of extrinsic antigens and in the subsequent initiation of an immune response.[6] HLA-DR molecules are expressed constitutively by B cells, activated T-cells, dendritic cells, macrophages and, at lower levels, by other cell types, such as endothelial and some epithelial cells, and their expression can be induced or increased by inflammatory stimuli, including cytokines.[7] A further mechanism allowing close cell-to-cell contact is dependent upon the expression of cell adhesion proteins, such as the intercellular adhesion molecule-1 (ICAM-1 or CD54), which belongs to the immunoglobulin superfamily. ICAM-1 is constitutively expressed by only a few cell types,[5,8] but its expression can be specifically upregulated by inflammatory mediators such as interferon-γ (IFN-γ), interleukin-1 (IL-1), and tumour necrosis factor–α (IFN-α).[5,7] Thus, it can be hypothesized that cells involved in inflammatory processes may express increased levels of HLA-DR and ICAM-1. Moreover, an increased expression of both HLA-DR and ICAM-1 molecules by bronchial epithelial cells has been reported in asthma and chronic bronchitis, and the extent of the expression of these markers seems to correlate with clinical parameters.[9] Since both asthma and chronic bronchitis are disorders characterized by the presence of activated mononuclear cells in the bronchial structures,[10] the present study was designed to evaluate whether mononuclear cells could increase the expression of HLA-DR and ICAM-1 molecules on bronchial epithelial cells. After this hypothesis proved to be correct, we tested the ability of nedocromil sodium (Tilade, Fisons plc, Loughborough, England), a topical anti-inflammatory drug used in patients with inflammatory airway disorders,

© 1994 Rapid Communications of Oxford Ltd and of a steroid, dexamethasone, to prevent the upregulation of HLA-DR and ICAM-1 expression in cultured human bronchial epithelial cells.

### Methods

*Reagents:* Laboratory of human carcinogenesis (LHC) basal medium, fetal calf serum (FCS), triiodothyronine (T3), hydrocortisone, and epinephrine were purchased from Biofluids Inc. (Rockville, MD, USA). RPMI-1640 (RPMI), phosphate buffered saline (PBS), DME, MEM, Hank's balanced salt solution (HBSS), streptomycin-penicillin, Ficoll, non-essential aminoacids, L-glutamine, trypan blue dye and fungizone were obtained from Flow ICN (Irvine, UK). Insulin, transferrin, bacterial type XIV protease, 2-mercaptoethanol, phytohaemoagglutinin (PHA), sodium azide and retinoic acid were purchased from Sigma Chemical Co. (St Louis, MO, USA). Phosphoethanolamine/ethanolamine, trace elements (selenium, manganese, molybdenum, vanadium, nickel, tin) and calcium were obtained from Fischer Scientific Co. (Pittsburgh, PA, USA). Frozen bovine pituitaries were obtained from Pel-Freez Biologicals (Rogers, AR, USA), and bovine type I collagen (Vitorgen 100) was obtained from Collagen Corp. (Palo Alto, CA, USA). Nedocromil sodium, as a pure powder, was a gift from Fisons Italchimici (Roma, Italy), dexamethasone was obtained from Collaborative Research Inc. (Bedford, MA, USA). Human recombinant IFN-γ was purchased from Technogenetics (Milano, Italy).

*Blood mononuclear cells collection:* Heparinized blood samples from three healthy donors were diluted 2:3 HBSS plus 0.35 g/l sodium bicarbonate, without calcium and magnesium. In order to isolate peripheral blood mononuclear cells (BMC), blood samples were layered on top of Ficoll gradients and centrifuged for 20 min at 1500 g, as described.[11] The BMC layer at the Ficoll interface (70–80% lymphocytes and 20–30% monocytes as determined by differential cell count) was gently aspirated and the cells washed, counted and resuspended at the desired density in complete medium (RPMI supplemented with 10% autologous serum, 2 g/l $NaHCO_3$, 50 U/ml penicillin, 50 μg/ml streptomycin, 2 mM L-glutamine, non-essential aminoacids and 5 mM 2-mercaptoethanol), The cells thus recovered were 98% viable, after a trypan blue dye exclusion test.

*Mononuclear cells stimulation:* The BMCs, $10^6$ cells/ml, were incubated at 37°C in 5% $Co_2$ with or without PHA (0.5 mg/ml). After three days, the supernatants were collected and stored at −80°C to be used later as condition medium (BMC-CM) in human bronchial epithelial cell (BEC) cultures, while the BMC were resuspended in complete medium plus 2.25 μl/ml of tritiated thymidine. After 18 h, the cells' proliferative response to PHA was assessed by measuring the 3HT uptake with a Beta counter.[11] Control wells included BMC cultures in complete medium, without mitogen.

*Human bronchial epithelial cells isolation:* Bronchial epithelial cells were prepared by a modification of the method described by Wu and colleagues,[12] as previously described.[13] Human bronchi, obtained at the time of surgical procedures, were trimmed of lung parenchyma and peribronchial vascular structures, and incubated at 4°C overnight in sterile calcium-free Eagle's MEM containing 0.1% bacterial protease (type XIV). The bronchial lumens were then gently rinsed several times with MEM containing 10% FCS to detach the epithelial cells. The collected cells were washed once in the same medium, filtered through one layer of surgical sterile gauze and washed twice with MEM. The cell count was manually determined using a standard haemocytometer. Extensive evaluation of cells prepared by this method has shown that > 95% of the harvested cells are epithelial cells (cytocheratine positive).[14]

In vitro *human bronchial epithelial cells culture:* BEC were plated for growth at 37°C in 5% $CO_2$ in 100 mm tissue culture plates (Becton Dickinson, Lincoln Park, NJ, USA) previously coated with bovine type I collagen. LHC basal medium[15] was used, supplemented with 5 μg/ml insulin, 10 μg/ml transferrin, 10 nM T3, 0.2 μM hydrocortisone, 0.33 nM retinoic acid, 5 μM phosphoethanolamine/ethanolamine, trace elements, 0.11 mM calcium, bovine pituitary extract[16] containing 10 mg/ml of protein (0.5%), 50 μg/ml penicillin-streptomycin, and 2 μg/ml fungizone. The LHC-supplemented medium was mixed 1:1 with RPMI, a solution that has been found to optimize BEC growth. In the average cell preparation, 20–30% of the viable cells plated at the start of the culture were attached to the tissue culture plates after 24 h and about a quarter of the adherent cells were able to proliferate. Because the confluent epithelial cells tend to assume a squamoid shape when replated and are less responsive to cytokines (O. Sacco, personal observation), the growing epithelial cells were continuously maintained in a sub-confluent state by replating the cells every 3–4 days.

*Blood mononuclear cell condition medium and IFN-γ effect on HLA-DR and ICAM-1 expression:* For the experiment, 30–40 × $10^3$ BEC/well were plated in 24-well tissue culture plates and stimulated with cytokines after 24 h. BMC-CM and human recombinant IFN-γ were used to evaluate the effects of a lymphocyte-derived cytokine on the expression of HLA-DR and ICAM-1 by BEC. In some experiments, BEC cultures were exposed to increasing concentrations of the BMC-CM or human recombinant IFN-γ. For control cultures, equivalent concentrations of 10% FCS in RPMI-1640 were added.

In some experiments, to test *in vitro* a possible inhibitory effect of corticosteroids and cromones on bronchial epithelial cell activation, bronchial epithelial cells were also exposed to dexamethasone and nedocromil sodium, at concentrations ranging from $10^{-4}$ to $10^{-9}$ M. For experiments performed with nedocromil and dexamethasone alone or together, bronchial epithelial cells were activated with 0.5% PHA-stimulated BMC supernatant, and the two drugs were added at the same time separately or together with the BMC stimulating supernatant. BECs were harvested for fluorescence study 36 h after exposure to BMC-CM.

*Evaluation of surface antigen expression:* The surface expression of HLA-DR and ICAM-1 was evaluated by specific antibody staining and flow-cytometry analysis. The BEC were trypsinized 36 h after stimulation with the BMC-CM, and then washed in MEM and resuspended in the staining medium which contained 1% fetal calf serum and 0.2% sodium azide in PBS. HBEC suspensions were placed into round-bottom microtitre 96 well/plates (Costar Corp., Cambridge, MA., USA) and incubated for 60 min at 4°C in 50 µl of the staining medium with a monoclonal antibody to human HLA-DR fluorescein isothiocyanate (FITC)-conjugate (Technogenetics, Milan, Italy) or a monoclonal mouse antibody anti-human ICAM-1 (CD54) (Sera Lab, Crawley Down, UK). A monoclonal antibody against human platelets, PTF 19, was used as a negative control preparation.[11] The cells stained with the anti-HLA-DR-FITC conjugated antibody were washed three times with staining medium, then fixed in PBS plus 1% paraformaldehyde (Eastman Kodak Co., Rochester, NY, USA). The cells incubated with the anti-ICAM-1 antibody were washed twice, stained with a goat anti-mouse-FITC conjugated antibody (Technogenetics srl, Milan, Italy) and then fixed as previously described. The cells were analysed by single colour immunofluorescence flow cytometry (FACS scan, Becton Dickinson Immunocytometry Systems, Mountain View, CA, USA). To compare the fluorescence intensities of different samples from the same experiments, the analyses were performed with identical settings of the logarithmic amplifier, and listmode files were analysed with Lysis II software (Becton Dickinson). After conversion to linear fluorescence intensity units to obtain a linear function or fluorescence intensity units over a wide range,[16] the average background linear fluorescence obtained with the control antibody level could be subtracted from the average fluorescence intensity of the specifically stained cells. The possible changes in cell size in the different experimental conditions were controlled by means of the forward light scatter signal of the flow cytometer, proportional to cell size and algebraically adjusted for increases in cell surface area, so that the intensity in relative linear fluorescence units could be obtained.[17] This correction allows the average fluorescence intensity to be directly correlated with the cell surface density of the stained antigens. All experiments were performed three times. To compare the drug downregulating effect on the expression of HLA-DR and ICAM-1 in different cell cultures, the average fluorescence intensity was transformed into relative fluorescence (control stimulated cells = 100% of relative fluorescence) and the drug effect was expressed as the percentage inhibition of the relative fluorescence.

$$\text{drug effect} = \frac{100 - (100 \times \text{average fluorescence of the sample})}{\text{average fluorescence of the control stimulated cells}}$$

*Statistical analysis:* All data are expressed as arithmetic mean ± standard error of the mean. Analysis of variance (ANOVA) was used to compare the potency of various concentrations of the drugs tested. The mean values were said to be statistically significant when the probability of the event was below 5% ($p < 0.05$).

### Results

*HLA-DR and ICAM-1 expression by human bronchial epithelial cells:* Control BEC, growing in LHC medium, show negligible basal expression of HLA-DR and ICAM-1 antigens. When incubated with human recombinant IFN-γ for 36 h at concentrations of 5, 10, 50, 200 and 1 000 U/ml, the expression of the two surface molecules was enhanced in a dose-dependent manner (Fig. 1A and 1B). Similarly, the addition of different concentrations of BMC-CM (0.5, 1 and 3%) to BEC induced, after 36 h, a dose-dependent increase of HLA-DR (Fig. 1) and ICAM-1 expression (Fig. 2), while the supernatant from the control (unstimulated BMCs) had no effect on the cell surface antigen expression. The relative fluorescence intensity related to the intensity of HLA-DR molecule expression showed a similarly close correlation with the concentration of IFN-γ ($p < 0.01$) and of the PHA-stimulated BMC-CM ($p < 0.01$) (Fig. 1). Using human recombinant IFN-γ as standard, we calculated that the IFN-γ-like activity in the BMC-CM was equivalent to 1.46 ± 0.54 units/µl of the human recombinant IFN-γ. A weaker correlation was found between the relative fluorescence intensity related to ICAM-1 molecule expression and either the concentration of IFN-γ ($p < 0.05$) or the BMC-CM concentration ($p < 0.05$) (Fig. 2).

*Pharmacologic inhibition of HLA-DR expression on bronchial epithelial cells:* When added to the BEC cultures at the same time as BMC-CM, nedocromil ($10^{-4}$ to $10^{-9}$ ) was able to inhibit the expression of HLA-DR on BEC in a dose-dependent manner (Fig. 3) at all the concentrations tested, and the effect was

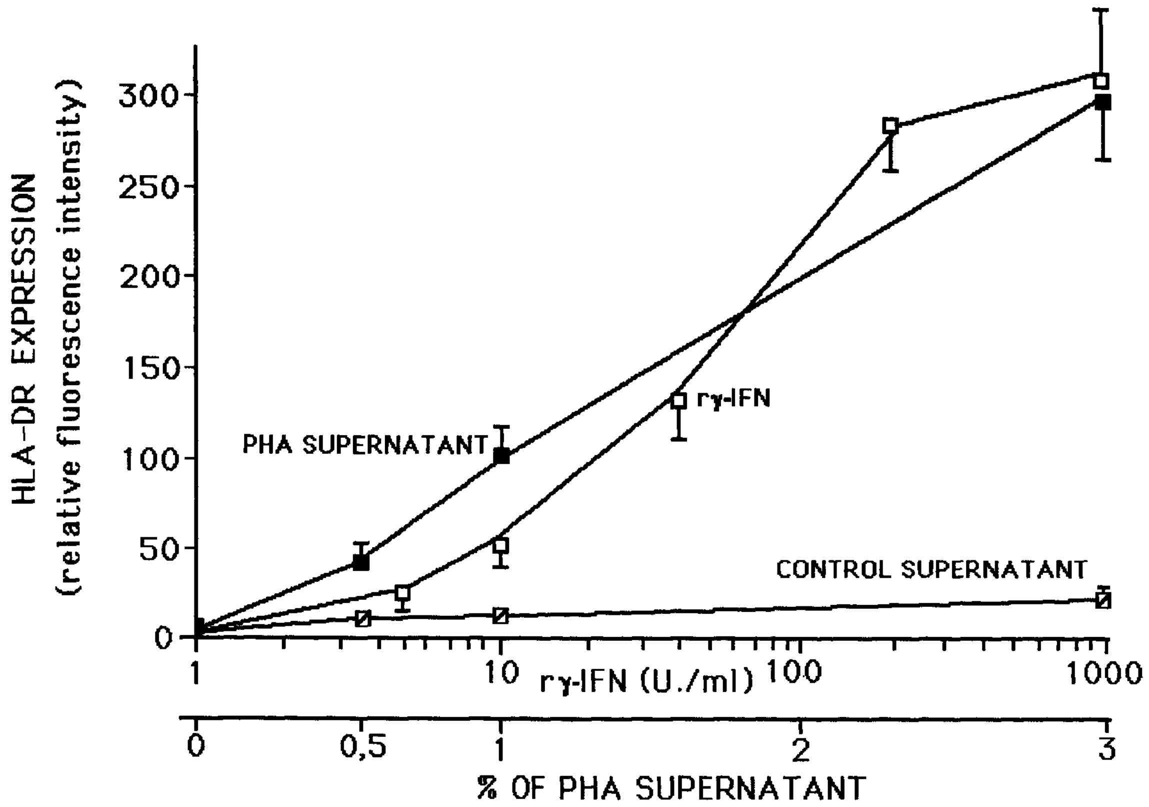

FIG. 1. Expression on human bronchial epithelial cells of HLA-DR surface antigens after 24 h incubation with medium alone (control supernatant), or with different concentrations of recombinant IFN-γ (rIFN-γ) or of PHA-activated mononuclear cell supernatant (PHA supernatant). The relative fluorescence intensity is expressed on the ordinate, while the concentrations of rIFN-γ or PHA supernatant are expressed on the abscissa.

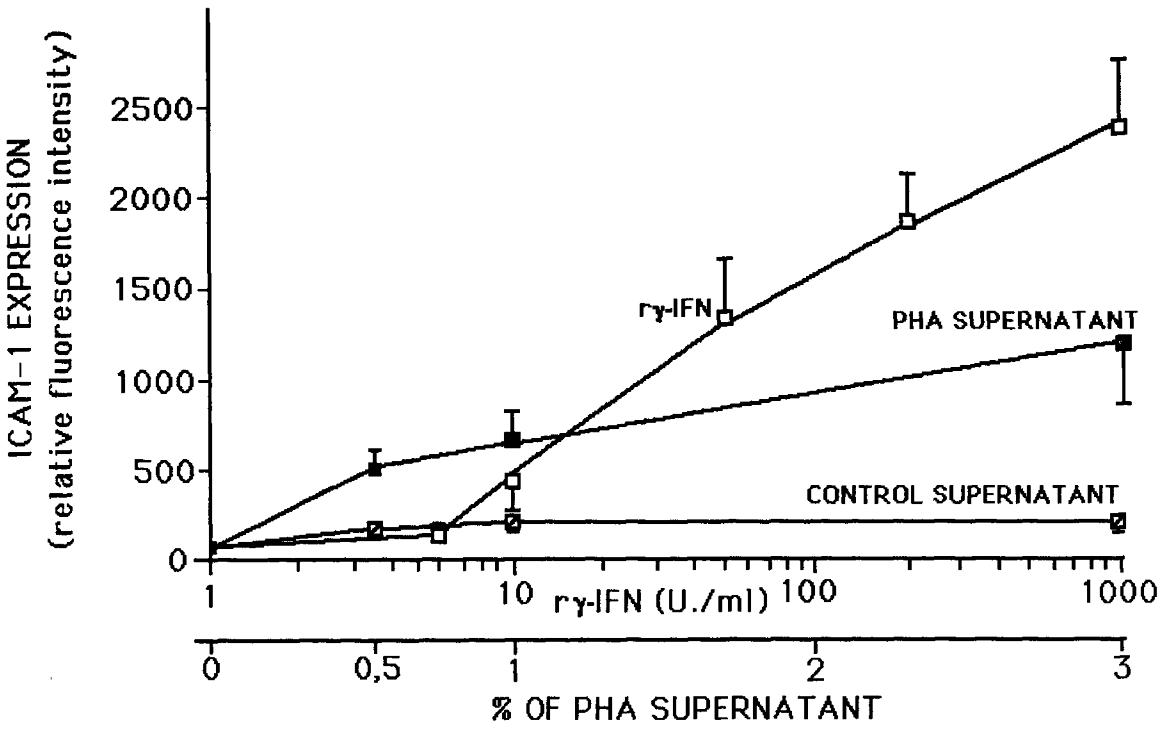

FIG. 2. Expression on human bronchial epithelial cells of ICAM-1 surface molecules after 24 h incubations with medium alone (control supernatant), or with different concentrations of recombinant IFN-γ (rIFN-γ) or of PHA-activated mononuclear cell supernatant (PHA supernatant). The relative fluorescence intensity is expressed on the ordinate, while the concentrations of rIFN-γ or PHA supernatant are expressed on the abscissa.

still statistically significant even at the lowest concentrations ($p < 0.05$, for all comparisons with BEC cultures without drugs). A dose-dependent, significant inhibition was also observed in cultures containing dexamethasone ($p < 0.05$, for all comparisons with BEC cultures without drugs). Nedocromil was more effective than dexamethasone at the highest concentrations tested ($10^{-4}$ to $10^{-6}$ M), but the differences were statistically significant only at $10^{-6}$ M ($p < 0.05$). The addition of both drugs to BEC cultures resulted in a synergistic downregulating effect only at the lowest concentrations tested, being statistically significant at $10^{-7}$ to $10^{-9}$ M ($p < 0.05$).

*Pharmacologic inhibition of ICAM-1 expression on bronchial epithelial cells:* When nedocromil and dexamethasone were added to the bronchial cell cultures together with the PHA-stimulated BMC supernatant, they were only able to downregulate ICAM-1 expression at concentrations between $10^{-4}$ and $10^{-7}$ M (Fig. 4). In contrast with the effect which had been observed on HLA-DR expression, dexamethasone had a significantly stronger effect when compared with nedocromil ($p < 0.05$) at all the concentrations which had proved to be effective (from $10^{-4}$ to $10^{-7}$ M). The two drugs together showed a significant synergistic effect at doses ranging from $10^{-6}$ to $10^{-9}$ M, and this was also observed at concentrations ($10^{-8}$ to $10^{-9}$ M) which had proved ineffective when both drugs were used alone (Fig. 4).

### Discussion

In this study, using BEC primary cultures, we have demonstrated that: (a) the expression of HLA-DR and ICAM-1 molecules on BEC can be upregulated by supernatants from BMC-CM as well as by IFN-γ; (b) nedocromil sodium shows a dose-dependent inhibitory effect on the expression of these activation surface molecules, this effect being stronger on HLA-DR than on ICAM-1 expression; (c) a similar dose-dependent inhibitory effect is observed using dexamethasone, which appears to be less active than nedocromil on HLA-DR expression and more active on ICAM-1 expression. HLA-DR molecules are expressed on the surface of a wide variety of cells belonging to the immune system, such as B lymphocytes, dendritic cells, monocytes, macrophages and activated T lymphocytes.[18–20] These surface molecules have been shown to play a key role in the interaction between antigen-presenting cells and T-lymphocytes which occurs during recognition of foreign antigens. In addition, HLA-DR molecules can also be expressed by cells that are not part of the immune system, such as endothelial cells and some epithelial cells, including human bronchial epithelial cells.[21–23] Although the functional implication of the expression of HLA-DR molecules by nonlymphoid tissues is not completely understood, it has been hypothesized that, at least for some organs, they might be involved in the active process

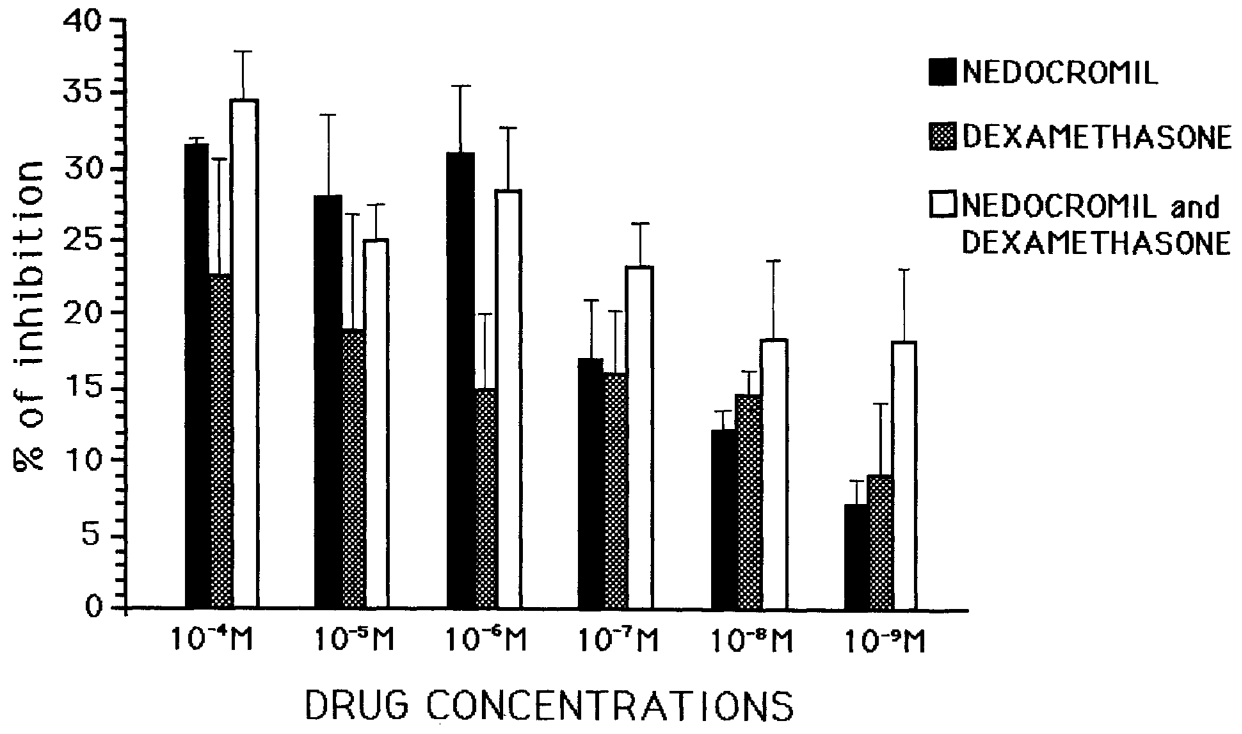

FIG. 3. Inhibitory effects of nedocromil sodium (■), dexamethasone (▩) or nedocromil sodium plus dexamethasone (□) on the expression of HLA-DR antigens by bronchial epithelial cells cultured for 24 h at 37°C, 5% $CO_2$ with 0.5% of supernatants from PHA-stimulated BMC. The percentage of inhibition is expressed on the ordinate, while the different concentrations of nedocromil sodium and dexamethasone are expressed on the abscissa.

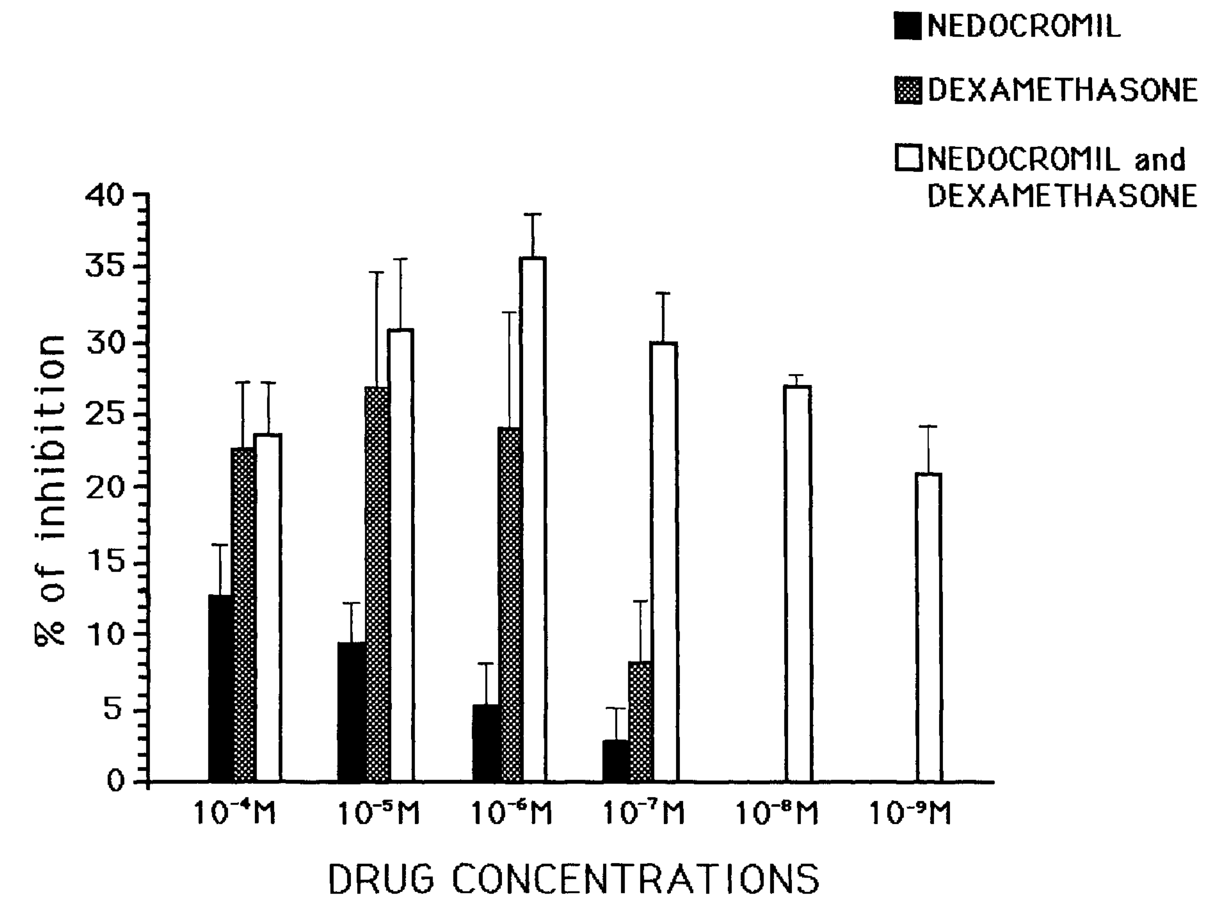

FIG. 4. Inhibitory effects of nedocromil sodium (■), demamethasone (▩), or nedocromil sodium plus dexamethasone (□) on the expression of ICAM-1 by bronchial epithelial cells cultured for 24 h at 37°C, 5% $CO_2$ with 0.5% of supernatants from PHA-stimulated BMC. The percentage of inhibition is expressed on the ordinate, while the different concentrations of nedocromil sodium and dexamethasone are expressed on the abscissa.

of antigen presentation and in the organization and the recruitment of inflammatory and immune effector cells. On the other hand, the ICAM-1 molecule belongs to the superfamily of the immunoglobulins[24] and acts as ligand for other (molecules expressed on monocytes and lymphocytes (LFA-1), and on neutrophils and eosinophils (Mac-1).[24,25] The expression of ICAM-1 is increased in sites of inflammatory reactions in tissues[26] and is associated with increased polymorphonuclear leukocyte (PMN) adhesion and activation.[27] Since PMN cytotoxicity is dependent, at least in part, on adhesion of PMNs to target cells, it is possible that this mechanism could be involved in the damage to the respiratory epithelium that is observed in airway inflammatory disorders characterized by the presence of neutrophils and eosinophils.[26] Therefore, it appears that the increased expression of HLA-DR and ICAM-1 may represent a mechanism by which BECs adhere to immunoeffector cells and deliver accessory signals. These mechanisms could be required for further T-cell activation, and for the recruitment of other cell types involved in inflammatory reactions, such as polymorphonuclear leukocytes and eosinophils.[26,27] In this context, it has been recently demonstrated that HBEC from asthmatic subjects and from patients with chronic bronchitis show increased ICAM-1 and HLA-DR expression, and that the levels of this expression correlate with the gravity of the illness.[9] The concept that both asthma and chronic bronchitis are characterized by the presence of activated mononuclear cells in the airways, and the evidence that activated BMC products activate BEC provided by the present study, give further support to the hypothesis that a complex cell-to-cell interaction occurs in inflammatory disorders of the airways.[9,10]

Since activated T-cells release great amounts of IFN-γ,[28] it is likely that this cytokine might have an important role in stimulating the expression of HLA-DR and ICAM-1 by bronchial epithelial cells. However, since HLA-DR expression is quite selectively upregulated by IFN-γ, while ICAM-1 molecules can be induced by different cytokines (mainly TNF-α and IL-1, besides IFN-γ), it is to be expected that a better correlation exists between the concentrations of BMC-CM and the relative fluorescence specific for HLA-DR, than has been found between BMC-CM and ICAM-1 as shown in the present study. It is also interesting that, like dexamethasone, nedocromil sodium, an anti-inflammatory drug used in the treatment of asthma and chronic bronchitis, is effective in downregulating the expression of HLA-DR and ICAM-1 on bronchial epithelial cells. Nedocromil sodium is thought to act on cells at the membrane level, where it inhibits the release of secretory mediators. This function is mediated through the activity of the drug on the $Cl^-$ channels, resulting in stabilization of the membrane.[29] It would be interesting to study whether the inhibitory effect of nedocromil sodium on the expression of HLA-DR and ICAM-1 on bronchial epithelial cells occurs not only at the cell membrane level but also at a transcriptional level, as demonstrated for corticosteroids.[30] The results of the present study demonstrate that it is possible to upregulate *in vitro* the expression of inflammatory markers on the BEC surface by the secretory products of activated lymphocytes. These results also add new insights into understanding the mechanisms that initiate and sustain the inflammatory processes in the lungs, and into understanding the effect at the cellular level of a drug already used in the treatment of the inflammatory reaction associated with asthma and chronic bronchitis. The observation that nedocromil and dexamethasone showed a sinergistic effect at the lowest concentrations tested on the expression of activation surface molecules by bronchial epithelial cells supports the concept of the 'steroid sparing effect' of cromones.

### References

1. Rennard SI, Beckmann JD, Robbins RA. Biology of airway epithelial cells. In: Crystal RG, West JB, eds. *The Lung: Scientific Foundations*. New York: Raven Press, 1991: 157–167.
2. Salari H, Chan-Yeung M. Arachidonic acid biosynthesis in human bronchial epithelial cells. *Am Rev Respir Dis* 1989; **19**: 635–643.
3. Shji S, Ertl RF, Linder J, Romberger DJ, Rennard SI. Bronchial epithelial cells produce chemotactic activity for bronchial epithelial cells. Possible role for fibronectin in airway repair. *Am Rev Respir Dis* 1990; **141**: 218–225.
4. Devalia JL, Campbell AM, Sapsford RJ, Runsznak C, Quint D, Godard P, Bousquet J, Davies RJ. Effect of nitrogen dioxide on synthesis of inflammatory cytokines expressed by human bronchial epithelial cells *in vitro*. *Am J Respir Cell Mol Biol* 1993; **9**: 271–278.
5. Montefort S, Holgate ST. Adhesion molecules and their role in inflammation. *Respir Med* 1991; **85**: 91–99.
6. Schawartz BD. HLA molecules: sentinel of the immune response. *Am J Resp Cell Mol Biol* 1991; **5**: 211–212.
7. Lee JS. Regulation of HLA class II gene expression. In: Albert ED, ed. *Immunobiology of HLA Vol 2*, New York: Springer-Verlag, 1989; **2**: 49–63.
8. Springer TA. Adhesion receptors of the immune system. *Nature* 1990; **346**: 425–434.
9. Vignola AM, Campbell AM, Chanez P, Bousquet J, Pascale P-L, Michel F-B, Godard P. HLA-DR and ICAM-1 expression on bronchial epithelial cells in asthma and chronic bronchitis. *Am Rev Respir Dis* 1993; **148**: 689–694.
10. Cosio MG, Ghezzo H, Hogg JC. The relations between structural changes in small airways and pulmonary function tests. *N Engl J Med* 1978; **298**: 1277–1281.
11. Rossi GA, Zocchi E, Sacco O, Balbi B, Ravazzoni C, Damiani G. Alveolar macrophage stimulation of T cell proliferation in autologous mixed lymphocyte reactions. Role of HLA-DR antigens. *Am Rev Respir Dis* 1986; **133**: 78–82.
12. Wu R, Smith D. Continuous multiplication of rabbit tracheal epithelial cells by transforming growth factor-beta. *Am J Respir Cell Mol Biol* 1982; **7**: 149–155.
13. Rossi GA, Sacco O, Balbi B, *et al.* Human ciliated bronchial epithelial cells: expression of the HLA-DR antigens and of the HLA-DR alpha gene, modulation of the HLA-DR antigens by gamma interferon and antigen presenting function in the mixed leucocyte reaction. *Am J Respir Cell Mol* 1990; **3**: 431–440.
14. Tazikawa H, Beckmann JD, Shoji S, *et al.* Pulmonary macrophages can stimulate cells growth bovine bronchial epithelial cells. *Am J Respir Cell Mol Biol* 1990; **2**: 245–255.
15. Lechner JF, La Vech MA. A serum-free method for culturing normal human bronchial epithelial cells and clonal density. *J Tissue Cult Meth* 1985; **9**: 43–48.
16. Muirhead KA, Schmitt TC, Muirhead AR. Determination of linear fluorescence intensities from flow cytometric data accumulated with logarithmic amplifiers. *Cytometry* 1983; **3**: 251–256.
17. Segal DM, Titus JA, Stephany DA. Fluorescence flow cytometry in the study of lymphoid cell receptors. *Methods Enzymol* 1987; **150**: 478–492.
18. Schawartz BD. HLA molecules: sentinel of the immune response. *Am J Respir Cell Mol Biol* 1991; **5**: 211–212.
19. Puri J, Taplits M, Alava M, Bonvini E, Hoffman V. Inhibition of release of arachidonic acid, superoxide, and IL-1 from human monocytes by monoclonal anti-HLA class II antibodies. *Inflammation* 1992; **16**: 31–43.
20. Kaufman JF, Auffray C, Korman AJ, Shackeldorf DA, Strominger J. The class II molecules of the human and murine major histocompatibility complex. *Cell* 1984; **36**: 1–13.
21. Wiman K, Curman B, Forsum U, *et al.* Occurrence of la antigens on tissue of non-lymphoid origin. *Nature* 1978; **276**: 711–713.
22. Daar AS, Fuggle SV, Fabre JW, Ting A, Morris PJ. The detailed distribution of MHC class II antigens in normal human organs. *Transplantation* 1984; **38**: 293–298.
23. Glanville AR, Tazelaar HD, Theodore J, *et al.* The distribution of MHC class I and II antigens on bronchial epithelium. *Am Rev Respir Dis* 1989; **139**: 330–334.
24. Montefort S, Roche WR, Howarth PH, *et al.* Intracellular adhesion molecule-1 (ICAM-1) and endothelial leukocyte adhesion molecule-1 (ELAM-1) expression in the bronchial mucosa of normal and asthmatic subjects. *Eur Respir J* 1992; **90**: 1379–1385.
25. Sacco O, Romberger D, Rizzino A, Beckman JD, Rennard SI, Spurzem JR. Spontaneous production of transforming growth factor-β2 by primary cultures of bronchial epithelial cells. *J Clin Inves* 1992; **90**: 1379–1385.
26. Tosi MF, Stark JM, Smith CW, Hamedani A, Gruenert DC, Infeld MD. Induction of ICAM-1 expression on human airway epithelial cells by inflammatory cytokines: effects on neutrophil-epithelial cells adhesion. *Am J Respir Cell Mol Biol* 1992; **7**: 214–221.
27. Smith CV. Molecular determinants of neutrophil adhesion. *Am J Respir Cell Mol Biol* 1990; **2**: 123–128.
28. Oddera S, Silvestri M, Sacco O, *et al.* The proliferative response to *Dermatophagoides Pteronyssinus* of blood T-cells is inhibited *in vitro* by budesonide. *Eur Respir J* 1992; **5**: 232s.
29. Reinsprecht M, Pecht I, Schindler H, Romanin C. Potent block of $Cl^-$ channels by antiallergic drugs. *Biochem Biophys Res Commun* 1992; **188**: 957–963.
30. Lantero S, Baldi L, Oddera S, *et al.* The release of chemotactic factor for eosinophils and of the synthesis and release of GM-CSF by allergen stimulated mononuclear cells are inhibited by budesonide. *Am Rev Respir Dis* 1994; **149**: A 943.

ACKNOWLEDGEMENTS. This work was supported in part by the Ricerca Finalizzata, 94, Ministero della Sanità, Rome. Vania Frangova is a recipient of a grant from the European Respiratory Society.
